# Study of Anisotropic Behavior in Sheet Metal Forming

**DOI:** 10.3390/ma17092031

**Published:** 2024-04-26

**Authors:** Haibo Wang, Qiang Niu, Yu Yan

**Affiliations:** School of Mechanical and Materials Engineering, North China University of Technology, Beijing 100144, China; wanghaibo@ncut.edu.cn (H.W.); nickyniu_work@163.com (Q.N.)

**Keywords:** anisotropy, yield function, three-dimensional stress state, finite element simulation, Yld2000-2d

## Abstract

Since sheet metal exhibits significant anisotropy in processing and forming, which has a significant impact on its performance during processing, forming, and use, we explore the anisotropic behavior of materials in the forming process of sheet metal. The ability of the Yld2000-2d criterion to describe anisotropic behavior is analyzed, and its accuracy for characterization of the anisotropic behavior of metal plates is improved, based on which anisotropic behavior is predicted in three-dimensional space. Theoretical and experimental results on the anisotropy of sheet metal are compared, and two materials, 5754O aluminum alloy and DP980 steel plate, are tested and analyzed, and the anisotropic behaviors, such as three-point bending and cylindrical deep-drawing, are well predicted.

## 1. Introduction

Sheet metals that have undergone remanufacturing and multiple rolling will present fibrous tissue or preferential orientation caused by crystallization, which will lead to anisotropy, meaning the material exhibits different mechanical properties in different directions. This anisotropic behavior has obvious influences on deformation during the forming process, such as earring and fracture on straight walls in deep drawing [1,2,3]. In general, the anisotropy of machined sheet metal and the resulting stresses and loose areas have a significant effect on its performance during forming and use, as in the following examples. (1) Mechanical property differences: anisotropy will lead to differences in the mechanical properties of the metal plate in different directions, such as strength, hardness, ductility, and so on. (2) Increased molding difficulty: anisotropy causes the deformation capacity in different directions to be different, which may lead to deformation in some directions in the molding process being difficult to achieve or requiring greater molding force. (3) Stress concentration: anisotropy makes it possible for stress concentration to occur when the metal plate is subjected to force, increasing the risk of local instability and fatigue damage. If the anisotropy is ignored, it may result in poorly formed or cracked aluminum sheets. If the anisotropic behavior during the sheet metal forming process can be predicted accurately, the forming quality can be greatly improved by utilizing the anisotropic characteristic effectively [4,5].

In order to accurately describe the anisotropic behavior of sheet metals, researchers all over the world have proposed many functions, such as Hill series yield functions [6,7,8], Hosford yield function [9], Barlat series yield function [10,11,12], and so on. Every yield function has its advantages and disadvantages. For example, the Hill48 yield function that is widely used in engineering applications is a simple function, the parameters of which are easy to solve, but its prediction of the anisotropic behavior of certain metals is not accurate enough [13]. In 1972, Hosford [14] proposed a yield function that can accurately describe the behaviors of cubic center and face center materials, but it does not include shear stress, which is inconvenient for engineering applications. In 2003, Barlat et al. [15] proposed a plane stress anisotropic yield function, which has high accuracy for anisotropic materials, especially for aluminum alloys [16,17]. The Yld2000-2d yield function has been widely used in the sheet metal forming field. At present, some special software already includes this yield function [18], while general software like ABAQUS6.4 needs to develop user subroutines to realize the application of the Yld2000-2d anisotropic yield function [19]. There are also some scholars who have developed anisotropic models for the anisotropy of a certain type of material; for example, Li et al. [20] developed a model that captures the tensile asymmetry of magnesium alloys, and Ji et al. [21] developed an anisotropic model that captures the anisotropy of aluminum alloys with respect to the strain rate. A generalized evolutionary plasticity model that takes into account thermal effects on the evolution of flow behavior was proposed by Lian et al. [22] to describe the temperature dependence of the anisotropic plastic flow behavior of the studied materials.

To accurately predict the plastic deformation behavior of materials in finite element simulations, it is necessary to use more accurate and convenient models [23,24,25]. In many cases, sheet metal experiences a three-dimensional stress state during the forming process, and the stress in the thickness direction will obviously affects the metal forming properties. Existing studies show that the stress in the thickness direction has obvious influences on sheet metal forming processes [26,27]. Existing studies also show that when compressive stress exists in the thickness direction, the forming limit curves will rise, and when tensile stress exists in the thickness direction, the forming limit curves will become lower [28,29]. In the sheet metal forming cases where the thickness stress is obvious, if the plane stress yield function is still used, the simulation accuracy might be decreased. Since some sheet metal forming simulations needs to consider the stress in the thickness direction, solid elements are more commonly adopted, and a 3D stress yield function should then be used.

Many three-dimensional stress anisotropic yield functions exist, among which the Hill48 and Yld2004-18p yield functions are widely accepted for engineering applications. Mu et al. [30] characterized the deformation behavior of DC06 steel plates more accurately by improving Hill48, and Rong et al. [31] used yld2004 to describe the thermal anisotropic behavior of AA7075 with high similarity. The Hill48 yield function, which has less flexibility, has low accuracy sometimes. It will lead to abnormal phenomena when describing aluminum alloy behaviors. The Yld2004-18p yield function has very high accuracy due to its high flexibility, which can describe the anisotropy of sheet metals very well. For example, the Yld2004 yield function can predict six and eight ears in deep drawing, while many other yield functions cannot [32]. The Yld2004 3D stress yield function has high accuracy, but the formula is complicated, and many experimental results are needed to determine the parameters’ values.

In many cases, the anisotropy of the sheet metal is relatively simple (for example, considering the anisotropic behaviors along three or four directions is enough), but 3D stress should be considered (i.e., the normal stress of sheet metal should not be neglected). Therefore, in order to better characterize the anisotropic behavior of sheet metal in practical machining applications, it is necessary to establish a yield function suitable for three-dimensional stress states so that a more convenient and accurate model can be constructed.

In this study, the anisotropic mechanical properties of two commonly used metal sheets (5754O and DP980) were analyzed. The Yld2000-2d yield function proposed by Barlat et al. [15] is extended into three-dimensional stress space for materials that are hydrostatic-stress-independent; this is called the Yld2000-3d yield function, and with it, the anisotropic properties of aluminum alloy plates under three-dimensional stress states are described more precisely. A finite element simulation of a three-point bending model of 5754O aluminum alloy sheet was performed by inserting the model into ABAQUS through the UMAT (VUMAT) subroutine. The accuracy of the new model in capturing the anisotropy of the aluminum alloy sheet is verified by comparing the simulation results with experimental results. In addition, deep drawing tests and finite element simulations of DP980 plates were conducted to further validate the generalizability of the method for characterizing the anisotropy of metal plates.

## 2. Sheet Anisotropy Characterization Methods

### 2.1. Yld2000-2d Yield Criterion Anisotropy Characterization Capability

The Yld2000-2d yield function [15] is expressed as:

(1) $$\phi \;=\phi^{\prime }+\phi^{\prime \prime }=2{\bar{\sigma }}^{m}$$

where

(2) $$\phi^{\prime }={\left|X_{1}^{\prime }-X_{2}^{\prime }\right|}^{m},\phi^{\prime \prime }={\left|2X_{2}^{\prime \prime }+X_{1}^{\prime \prime }\right|}^{m}+{\left|2X_{1}^{\prime \prime }+X_{2}^{\prime \prime }\right|}^{m}$$

In Equation (1), ϕ is the sum of the two isotropic functions. In Equation (2), $X_{i}^{\prime }$ and $X_{j}^{\prime \prime }$ are the principal values of the matrices $X^{\prime }$ and $X^{\prime \prime }$:

(3) $$\{\begin{array}{c} X_{1}^{\prime }=\frac{1}{2}(X_{11}^{\prime }+X_{22}^{\prime }+\sqrt{{\left(X_{11}^{\prime }-X_{22}^{\prime }\right)}^{2}+4{X_{12}^{\prime }}^{2}})\\ X_{2}^{\prime }=\frac{1}{2}(X_{11}^{\prime }+X_{22}^{\prime }-\sqrt{{\left(X_{11}^{\prime }-X_{22}^{\prime }\right)}^{2}+4{X_{12}^{\prime }}^{2}})\\ X_{1}^{\prime \prime }=\frac{1}{2}(X_{11}^{\prime \prime }+X_{22}^{\prime \prime }+\sqrt{{\left(X_{11}^{\prime \prime }-X_{22}^{\prime \prime }\right)}^{2}+4{X_{12}^{\prime \prime }}^{2}})\\ X_{2}^{\prime \prime }=\frac{1}{2}(X_{11}^{\prime \prime }+X_{22}^{\prime \prime }-\sqrt{{\left(X_{11}^{\prime \prime }-X_{22}^{\prime \prime }\right)}^{2}+4{X_{12}^{\prime \prime }}^{2}}) \end{array}$$

The elements of $X^{\prime }$ and $X^{\prime \prime }$ are converted from Cauchy stress:

(4) $$X^{\prime }=L^{\prime }\sigma ,\;X^{\prime \prime }=L^{\prime \prime }\sigma$$

where

(5) $$\left[\begin{array}{c} L_{11}^{\prime }\\ L_{12}^{\prime }\\ L_{21}^{\prime }\\ L_{22}^{\prime }\\ L_{66}^{\prime } \end{array}\right]=\left[\begin{array}{ccc} 2/3 & 0 & 0\\ -1/3 & 0 & 0\\ 0 & -1/3 & 0\\ 0 & 2/3 & 0\\ 0 & 0 & 1 \end{array}\right]\cdot \left[\begin{array}{c} \alpha_{1}\\ \alpha_{2}\\ \alpha_{7} \end{array}\right]$$

(6) $$\left[\begin{array}{c} L_{11}^{\prime \prime }\\ L_{12}^{\prime \prime }\\ L_{21}^{\prime \prime }\\ L_{22}^{\prime \prime }\\ L_{66}^{\prime \prime } \end{array}\right]=\frac{1}{9}\cdot \left[\begin{array}{ccccc} -2 & 2 & 8 & -2 & 0\\ 1 & -4 & -4 & 4 & 0\\ 4 & -4 & -4 & 1 & 0\\ -2 & 8 & 2 & -2 & 0\\ 0 & 0 & 0 & 0 & 9 \end{array}\right]\cdot \left[\begin{array}{c} \alpha_{3}\\ \alpha_{4}\\ \alpha_{5}\\ \alpha_{6}\\ \alpha_{8} \end{array}\right]$$

where *m* is the material parameter, and $\alpha_{1}-\alpha_{8}$ are the eight anisotropic parameters of the anisotropic yield function, which can be calculated with the material properties. When the eight parameters are equal to 1, the function will return to the isotropic function.

### 2.2. Prediction of Anisotropic Behavior of Materials in Three-Dimensional Stress State

As stated above, the stress components included in the Yld2000-2d yield function are $\sigma_{xx}$, $\sigma_{yy}$, and $\sigma_{xy}$ in the X-Y plane. In many cases, however, the normal stress and shear stress in the thickness direction cannot be neglected and have a significant effect on the sheet metal forming process. In this study, the Yld2000-2d yield function is modified, and the normal stress along the thickness direction is included firstly. That is, the Yld2000-2d yield function for the 2D case is extended into three-dimensional stress space. 

**Figure 1:**
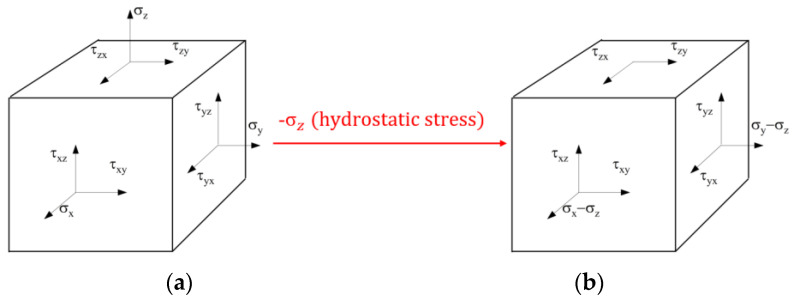
Stress state analysis of the one material point: (**a**) three-dimensional stress; (**b**) equivalent two-dimensional stress.

As shown in Figure 1a, the material point is subjected to different stresses in different directions and is in equilibrium. Convert the 3D stress state of Figure 1a by subtracting a hydrostatic stress equal to the algebraic value of thickness direction stress $\sigma_{z}$ (adding a hydrostatic stress, the algebraic value of which is $-\sigma_{z}$). The converted stress state is shown in Figure 1b. According to the continuity hypothesis, for the hydrostatic-stress-independent materials, the hydrostatic stress will only cause elastic volume changes, and will not affect the plastic deformation rules. Therefore, the plastic deformation caused by the two stress states shown in Figure 1 will be completely the same.

As for the normal stress, the above conversion expression from the three-dimensional to two-dimensional stress space is:

(7) $$\Omega :\;R^{3}\to R^{2},\left(\begin{array}{c} \sigma_{xx}\\ \sigma_{yy}\\ \sigma_{zz} \end{array}\right)\mapsto \left(\begin{array}{c} \sigma_{xx}-\sigma_{zz}\\ \sigma_{yy}-\sigma_{zz} \end{array}\right)\mapsto \left(\begin{array}{c} \Sigma_{1}\\ \Sigma_{2} \end{array}\right)=\left(\begin{array}{c} \sigma_{xx}-\sigma_{zz}\\ \sigma_{yy}-\sigma_{zz} \end{array}\right)=\left(\begin{array}{c} \sigma_{xx}^{\prime }\\ \sigma_{yy}^{\prime } \end{array}\right)$$

Besides the three normal stresses $\sigma_{x},\;\;\;\;\sigma_{y}$, and $\;\sigma_{z}$, the three-dimensional stress components also include the three shear stress components $\sigma_{xy},\;\;\;\;\sigma_{yz}$, and $\;\sigma_{zx}$. The shear stresses $\sigma_{yz}$ and $\;\;\sigma_{zx}$ are handled with the method that was used for $\sigma_{xy}$ in the Yld2000-2d yield function.

Thus, the Yld2000-2d yield function is extended into three-dimensional stress space and is named “Yld2000-3d” in this study, the expression of which is

(8) $$\phi =\phi^{\prime }+\phi^{\prime \prime }=2{\bar{\sigma }}^{m}$$

where

(9) $$\phi^{\prime }={\left|X_{1}^{\prime }-X_{2}^{\prime }\right|}^{m},\phi^{\prime \prime }={\left|2X_{2}^{\prime \prime }+X_{1}^{\prime \prime }\right|}^{m}+{\left|2X_{1}^{\prime \prime }+X_{2}^{\prime \prime }\right|}^{m}$$

(10) $$\{\begin{array}{c} X_{1}^{\prime }=\frac{1}{2}(X_{11}^{\prime }+X_{22}^{\prime }+\sqrt{{\left(X_{11}^{\prime }-X_{22}^{\prime }\right)}^{2}+4{\tilde{X}}^{\prime 2}})\\ X_{2}^{\prime }=\frac{1}{2}(X_{11}^{\prime }+X_{22}^{\prime }-\sqrt{{\left(X_{11}^{\prime }-X_{22}^{\prime }\right)}^{2}+4{\tilde{X}}^{\prime 2}})\\ X_{1}^{\prime \prime }=\frac{1}{2}(X_{11}^{\prime \prime }+X_{22}^{\prime \prime }+\sqrt{{\left(X_{11}^{\prime \prime }-X_{22}^{\prime \prime }\right)}^{2}+4{\tilde{X}}^{\prime \prime 2}})\\ X_{2}^{\prime \prime }=\frac{1}{2}(X_{11}^{\prime \prime }+X_{22}^{\prime \prime }-\sqrt{{\left(X_{11}^{\prime \prime }-X_{22}^{\prime \prime }\right)}^{2}+4{\tilde{X}}^{\prime \prime 2}}) \end{array}$$

where

(11) $${\tilde{X}}^{\prime 2}=X_{12}^{\prime 2}+X_{23}^{\prime 2}+X_{13}^{\prime 2}$$

(12) $${\tilde{X}}^{\prime \prime 2}=X_{12}^{\prime \prime 2}+X_{23}^{\prime \prime 2}+X_{13}^{\prime \prime 2}$$

The components of $X^{\prime }$ and $X^{\prime \prime }$ are converted from the Cauchy stress as follows:

(13) $$X^{\prime }=\left[\begin{array}{c} X_{11}^{\prime }\\ X_{22}^{\prime }\\ X_{12}^{\prime }\\ X_{23}^{\prime }\\ X_{13}^{\prime } \end{array}\right]=\left[\begin{array}{ccccc} L_{11}^{\prime } & L_{12}^{\prime } & 0 & 0 & 0\\ L_{21}^{\prime } & L_{22}^{\prime } & 0 & 0 & 0\\ 0 & 0 & L_{44}^{\prime } & 0 & 0\\ 0 & 0 & 0 & L_{55}^{\prime } & 0\\ 0 & 0 & 0 & 0 & L_{66}^{\prime } \end{array}\right]\cdot \left[\begin{array}{c} \sigma_{xx}-\sigma_{zz}\\ \sigma_{yy}-\sigma_{zz}\\ \sigma_{xy}\\ \sigma_{yz}\\ \sigma_{xz} \end{array}\right]$$

(14) $$X^{\prime \prime }=\left[\begin{array}{c} X_{11}^{\prime \prime }\\ X_{22}^{\prime \prime }\\ X_{12}^{\prime \prime }\\ X_{23}^{\prime \prime }\\ X_{13}^{\prime \prime } \end{array}\right]=\left[\begin{array}{ccccc} L_{11}^{\prime \prime } & L_{12}^{\prime \prime } & 0 & 0 & 0\\ L_{21}^{\prime \prime } & L_{22}^{\prime \prime } & 0 & 0 & 0\\ 0 & 0 & L_{44}^{\prime \prime } & 0 & 0\\ 0 & 0 & 0 & L_{55}^{\prime \prime } & 0\\ 0 & 0 & 0 & 0 & L_{66}^{\prime \prime } \end{array}\right]\cdot \left[\begin{array}{c} \sigma_{xx}-\sigma_{zz}\\ \sigma_{yy}-\sigma_{zz}\\ \sigma_{xy}\\ \sigma_{yz}\\ \sigma_{xz} \end{array}\right]$$

From Equations (13) and (14), it can be seen that in the yield function expression, the coefficients of the three shear stress components $\sigma_{xy},\;\sigma_{yz}$, and $\sigma_{zx}$ are $L_{44}^{\prime }$, $L_{55}^{\prime }$, $L_{66}^{\prime }$ and $L_{44}^{\prime \prime }$, $L_{55}^{\prime \prime }$, $L_{66}^{\prime \prime }$, which represent the shear anisotropic properties and can be expressed as

(15) $$\;\left[\begin{array}{c} L_{11}^{\prime }\\ L_{12}^{\prime }\\ L_{21}^{\prime }\\ L_{22}^{\prime }\\ \begin{array}{l} L_{44}^{\prime }\\ L_{55}^{\prime }\\ L_{66}^{\prime } \end{array} \end{array}\right]=\left[\begin{array}{l} \;\;\;2/3\;\;\;\;\;\;\;\;\;\;\;\;\;\;\;\;\;\;0\;\;\;\;\;\;\;\;\;\;\;\;\;\;0\;\;\;\;\;\;\;\;\;\;\;\;0\;\;\;\;\;\;\;\;\;0\\ -1/3\;\;\;\;\;\;\;\;\;\;\;\;\;\;\;\;\;\;0\;\;\;\;\;\;\;\;\;\;\;\;\;\;0\;\;\;\;\;\;\;\;\;\;\;\;0\;\;\;\;\;\;\;\;\;0\\ \;\;\;\;\;0\;\;\;\;\;\;\;\;\;\;\;\;\;-1/3\;\;\;\;\;\;\;\;\;\;\;\;0\;\;\;\;\;\;\;\;\;\;\;\;0\;\;\;\;\;\;\;\;\;0\\ \;\;\;\;\;0\;\;\;\;\;\;\;\;\;\;\;\;\;\;\;\;\;\;2/3\;\;\;\;\;\;\;\;\;\;\;\;0\;\;\;\;\;\;\;\;\;\;\;\;0\;\;\;\;\;\;\;\;\;0\\ \;\;\;\;\;0\;\;\;\;\;\;\;\;\;\;\;\;\;\;\;\;\;\;\;\;0\;\;\;\;\;\;\;\;\;\;\;\;\;\;1\;\;\;\;\;\;\;\;\;\;\;\;0\;\;\;\;\;\;\;\;\;0\\ \;\;\;\;\;0\;\;\;\;\;\;\;\;\;\;\;\;\;\;\;\;\;\;\;\;0\;\;\;\;\;\;\;\;\;\;\;\;\;\;0\;\;\;\;\;\;\;\;\;\;\;\;1\;\;\;\;\;\;\;\;\;0\\ \;\;\;\;\;0\;\;\;\;\;\;\;\;\;\;\;\;\;\;\;\;\;\;\;\;0\;\;\;\;\;\;\;\;\;\;\;\;\;\;0\;\;\;\;\;\;\;\;\;\;\;\;0\;\;\;\;\;\;\;\;\;1 \end{array}\right]\cdot \left[\begin{array}{l} \alpha_{1}\\ \alpha_{2}\\ \alpha_{7}\\ \alpha_{9}\\ \alpha_{10} \end{array}\right]$$

(16) $$\left[\begin{array}{c} L_{11}^{\prime \prime }\\ L_{12}^{\prime \prime }\\ L_{21}^{\prime \prime }\\ L_{22}^{\prime \prime }\\ \begin{array}{l} L_{44}^{\prime \prime }\\ L_{55}^{\prime \prime }\\ L_{66}^{\prime \prime } \end{array} \end{array}\right]=\frac{1}{9}\cdot \left[\begin{array}{ccccccc} -2 & 2 & 8 & -2 & 0 & 0 & 0\\ 1 & -4 & -4 & 4 & 0 & 0 & 0\\ 4 & -4 & -4 & 1 & 0 & 0 & 0\\ -2 & 8 & 2 & -2 & 0 & 0 & 0\\ 0 & 0 & 0 & 0 & 9 & 0 & 0\\ 0 & 0 & 0 & 0 & 0 & 9 & 0\\ 0 & 0 & 0 & 0 & 0 & 0 & 9 \end{array}\right]\cdot \left[\begin{array}{c} \alpha_{3}\\ \alpha_{4}\\ \alpha_{5}\\ \alpha_{6}\\ \begin{array}{l} \alpha_{8}\\ \alpha_{11}\\ \alpha_{12} \end{array} \end{array}\right]$$

where $\alpha_{1}$–$\alpha_{12}$ are anisotropic material parameters.

Because the material property data in the XZ and YZ planes are difficult to obtain in basic sheet metal tests, the shear isotropy is assumed in this study, namely the anisotropic coefficients for $\sigma_{xy},\;\;\;\;\sigma_{yz},\;\;\;\;\sigma_{zx}$ are assumed to be the same. Liu and Raemy used similar methods in their yield functions [33,34]. Therefore, in Equations (13) and (14), we have $L_{44}^{\prime }=L_{55}^{\prime }=L_{66}^{\prime }$ and $L_{44}^{\prime \prime }=L_{55}^{\prime \prime }=L_{66}^{\prime \prime }$. In other words, we have $\alpha_{7}=\alpha_{9}=\alpha_{10}$ and $\alpha_{8}=\alpha_{11}=\alpha_{12}$. Surely, the shear anisotropy can be represented if corresponding material properties in the XZ and YZ planes can be obtained.

It is shown that the developed yield function still used the linear transformation method. Yoshida et al. [35] perform the same conversions on a six-order polynomial, and the convexity of a certain cross-section of the three-dimensional yield function is considered. In this study, the three-dimensional stress yield function developed can ensure strict convexity. It is easy to prove that its convexity is in consistency with the original Yld2000-2d yield function. Besides the uniaxial and the biaxial tensile mechanical properties, the thickness stress components $\sigma_{zz},\;\;\;\;\sigma_{yz},\;\;\;\;\sigma_{zx}$ are also included in the Yld2000-3d yield function. On the other hand, from Equations (13) and (14), it is easy to find that the Yld2000-3d yield function only considers the value of the thickness stress and does not consider the anisotropy of the thickness stress. Cazacu [36] found that in the sheet-forming processes, the existence of the thickness stress should be considered, but the anisotropy of the thickness direction stress does not need to be considered.

The determination of the parameters is an important step for the application of the developed Yld2000-3d yield function. Comparing it with Yld2000-2d yield function, no more parameters are included in the Yld2000-3d function, and the determining method in this study is exactly the same as that of the original Yld2000-2d yield function [16] (only some anisotropic properties under planes stress state are needed), so it will not be presented in detail. As a result, the anisotropy in the metal sheet-forming process can be captured more conveniently and accurately for practical engineering use.

## 3. Results and Discussion

### 3.1. Experimental and Material Data

**Figure 2:**
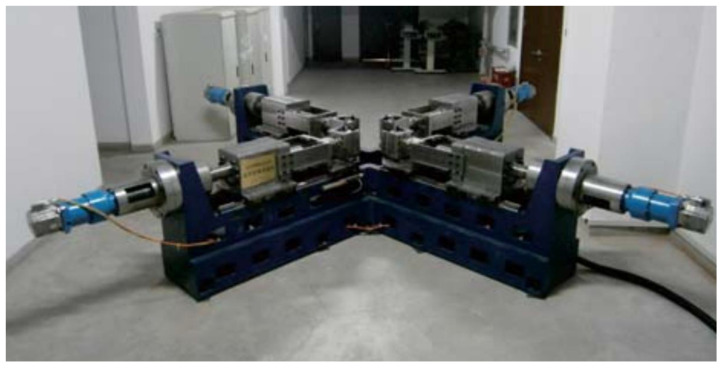
Biaxial loading test machine.

Biaxial tensile testing of cruciform specimens was performed on an established biaxial tensile testing system. The test system is modified and improved from the biaxial loading test machine shown in Figure 2, which can realize the synergistic motion of four axes in two directions and can carry out biaxial tensile tests under different proportional loading paths. The characteristics of the test system are as follows: tensile speed 0.6–6 mm/min, force transducer accuracy ≤2%, chuck maximum stroke 400 mm, working environment temperature −10–50 °C. It can be seen that the bi-directional tensile test system meets the needs of the test in terms of requirements for precision, reliability, and accuracy.

Biaxial tensile testing of cruciform specimens is usually performed using both load-controlled and strain-controlled methods. The study of yielding behavior is generally carried out in stress space, and the yield criterion is usually expressed in terms of stresses; hence, the load control approach is used in this paper. For the cruciform specimen, a 5754O (t = 1 mm) aluminum alloy plate with r < 1 was selected. The test specimens were prepared by laser cutting, and the specific dimensions were obtained from Wang et al.’s paper [19].

Before the test, the extensometer for strain measurement and the force transducer for load measurement were calibrated, and the calibration errors were less than 1%. During the test, the main control direction tensile speed is 2 mm/min, the initial measurement area of the strain in the center area is 50 mm × 50 mm, and the strain value during deformation is measured by an extensometer installed on both surfaces of the specimen at a distance of 50 mm, respectively. The measurement range of the extensometer is 10 mm, and the pins of the extensometer are fixed to the specimen by clamping during the measurement.

The ratios of the rolling direction to the load perpendicular to the rolling direction used in the test were 4:0, 4:1, 4:2, 4:3, 4:4, 3:4, 2:4, 1:4, and 0:4, respectively. The test is stopped when the load curve decreases or when rupture of the specimen is observed, and the data collected in the test are the load on the cross arm of the specimen and the deformation in the center area. Each set of data was repeated three times, and its average value was finally solved to obtain the mechanical properties of 5754O, as shown in Table 1 and Table 2.

### 3.2. Three-Dimensional Stress State Material Anisotropy

The Yld2000-2d yield function can accurately predict the mechanical behaviors of 5754O aluminum alloy [37,38] from existing research results. In this study, considering the normal stress and shear stress in the sheet thickness direction, 5754O aluminum alloy is adopted as an example to verify the accuracy of the developed Yld2000-3d yield function. The uniaxial and biaxial tensile properties of 5754O aluminum alloy are shown in Table 1 and Table 2, where β represents the plastic strain direction under plane stress condition, and the r-value is the coefficient of anisotropy in the thickness direction. They have the following definitions:

(17) $$\beta =\arctan(d\varepsilon_{y}/d\varepsilon_{x})$$

(18) $$r=d\varepsilon_{y}/d\varepsilon_{z}$$

By the principal of volume invariance, $d\varepsilon_{x}+d\varepsilon_{y}+d\varepsilon_{z}=0$, and the r-value and β can be transformed into each other computationally.

According to the parameter determination method of Yld2000-3d, which is the same as that of the Yld2000-2d yield function, the material constants of 5754O aluminum alloy sheets are obtained as:

$$\alpha_{1}=0.9983\;\alpha_{2}=0.9323\;\alpha_{3}=0.9827\;\alpha_{4}=0.9675$$

$$\alpha_{5}=1.0109\;\alpha_{6}=0.9827\;\alpha_{7}=1.0484\;\alpha_{8}=0.8638$$

**Figure 3:**
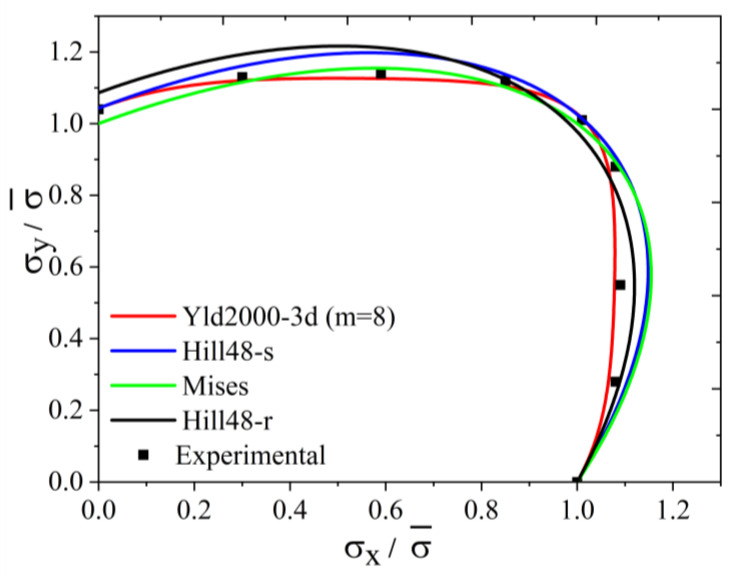
The normalized plastic work contours in $\sigma_{x}-\sigma_{y}$ plane of 5754O aluminum alloy sheet based on different yield functions.

**Figure 4:**
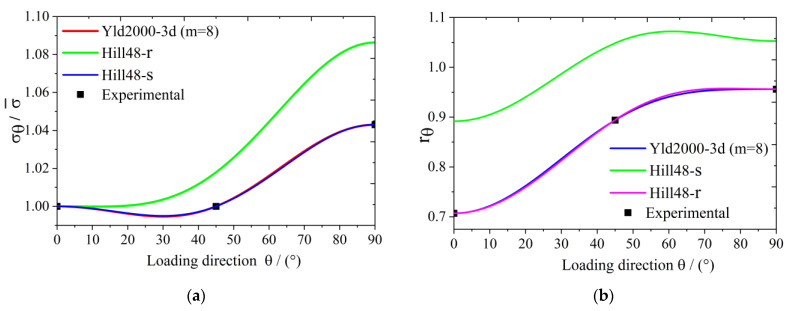
The uniaxial tensile properties of 5754O aluminum alloy sheet predicted based on different yield functions: (**a**) stress prediction; (**b**) *r* value prediction.

**Figure 5:**
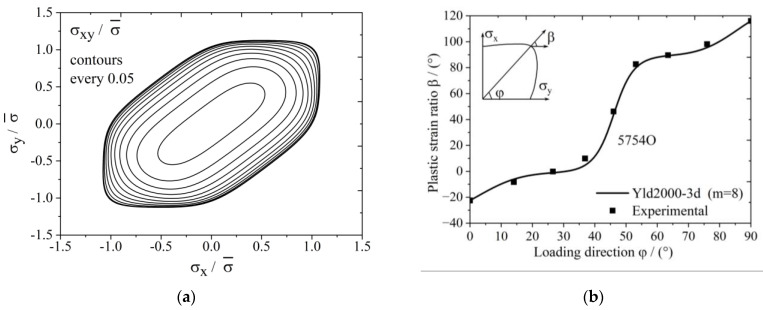
Tricomponent yield surface of Yld2000-3d for 5754O in $\sigma_{x}-\sigma_{y}$ plane: (**a**) yield surfaces; (**b**) plastic strain ratio under different loading.

**Figure 6:**
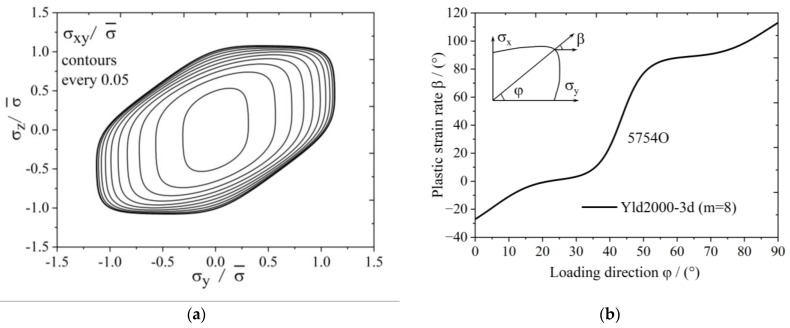
Tricomponent yield surface of Yld2000-3d for 5754O in $\sigma_{y}-\sigma_{z}$ plane: (**a**) yield surfaces; (**b**) plastic strain ratio under different loading.

**Figure 7:**
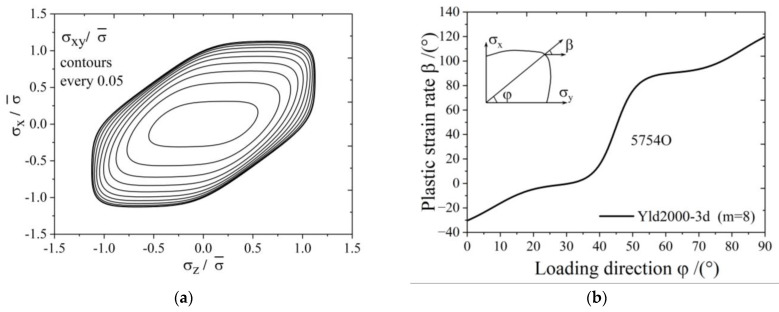
Tricomponent yield surface of Yld2000-3d for 5754O in $\sigma_{z}-\sigma_{x}$ plane: (**a**) yield surfaces; (**b**) plastic strain ratio under different loading.

The yield surface of 5754O aluminum sheet represented by the Yld2000-3d yield function is shown in Figure 3. Figure 4a,b show the uniaxial tensile anisotropy of a 5754O aluminum sheet predicted by the Yld2000-3d yield function. From the theoretical and experimental results, it is shown that the Yld2000-3d (Yld2000-2d) yield function can accurately characterize the uniaxial and biaxial tensile properties of a 5754O aluminum alloy sheet. The tricomponent yield surfaces in the $\sigma_{x}-\sigma_{y}$, $\sigma_{y}-\sigma_{z}$, and $\sigma_{z}-\sigma_{x}$ stress space based on the Yld2000-3d yield function are shown in Figure 5a, Figure 6a and Figure 7a, respectively. The results showed that the tricomponent yield surfaces of 5754O aluminum sheet in different stress spaces are very different from each other due to anisotropy. Figure 5b is the plastic strain direction in $\sigma_{x}-\sigma_{y}$ stress space, where $\varphi =\arctan(\sigma_{y}/\sigma_{x})$ is the loading angle. It is shown that the plastic strain direction for different loading angle agrees very well with the experimental results, which further verifies the anisotropic description ability of the Yld2000-3d (Yld2000-2d) yield function. Figure 6b and Figure 7b are the plastic strain direction in $\sigma_{y}-\sigma_{z}$ and $\sigma_{z}-\sigma_{x}$ stress space predicted by the Yld2000-3d yield function, respectively. 

### 3.3. 5754O Aluminum Alloy Plate Anisotropy Prediction Verification

In this study, the complete implicit back-Euler integration mapping algorithm is adopted to implement the developed Yld2000-3d yield function in ABAQUS commercial software since it has been used successfully for the implementation of Yld2000-2d in ABAQUS [39]. The specific stress transfer process in the subroutine is derived in detail in Appendix A.

**Figure 8:**
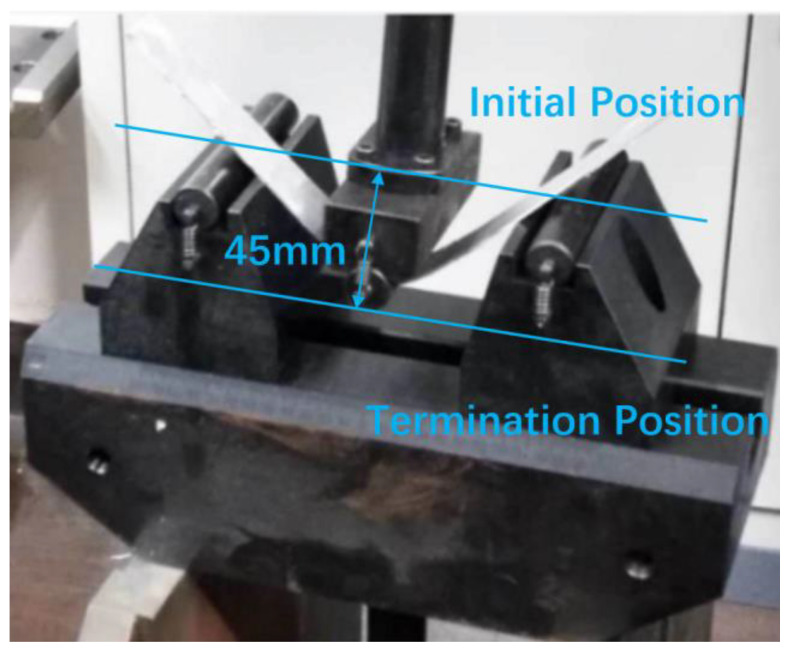
Three-point bending equipment.

The three-point bending experiments’ equipment is shown in Figure 8. A 5754O aluminum alloy sheet is adopted in the experiment, the properties of which are described in Section 2.1. The specimen is 300 mm in length, 30 mm in width, and 2 mm in thickness. The diameter of the upper die and lower die are both 20 mm, and the die gap is 150 mm. The punch displacement is 45 mm. 

**Figure 9:**
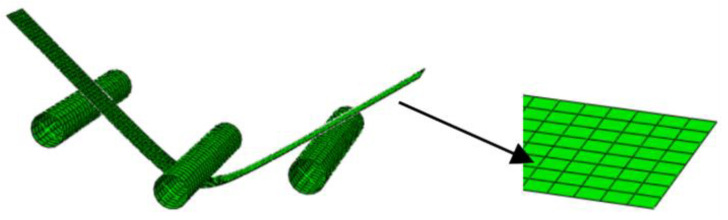
FEM model established using S4R shell elements with five and seven integration points.

**Figure 10:**
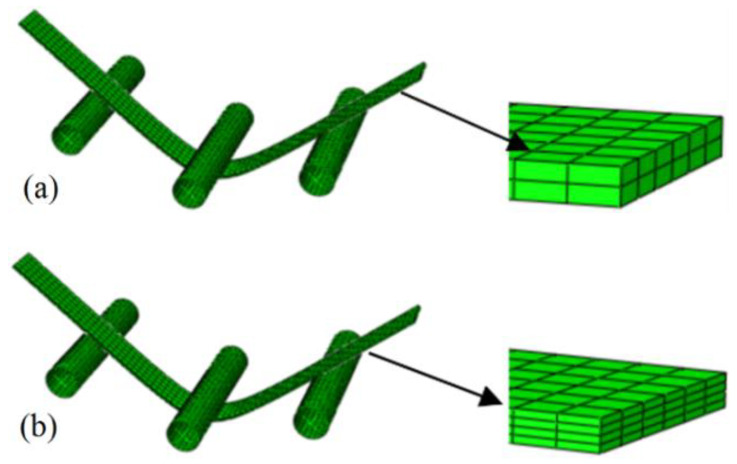
FEM model established using C3D8R solid elements with two and four layers of elements: (**a**) two layers of elements; (**b**) four layers of elements.

In the FEM simulations, the two-dimensional S4R shell elements and three-dimensional C3D8R solid elements are used based on the Yld2000-2d yield function and the Yld2000-3d yield function, respectively. The dies are established with discrete rigid elements, limiting the movement of the three-point-bending thin plate specimen in the x-direction (Ux) and the rotation in both directions of the y-axis and z-axis (URy, URz). The FEM model is shown in Figure 9 and Figure 10. 

**Figure 11:**
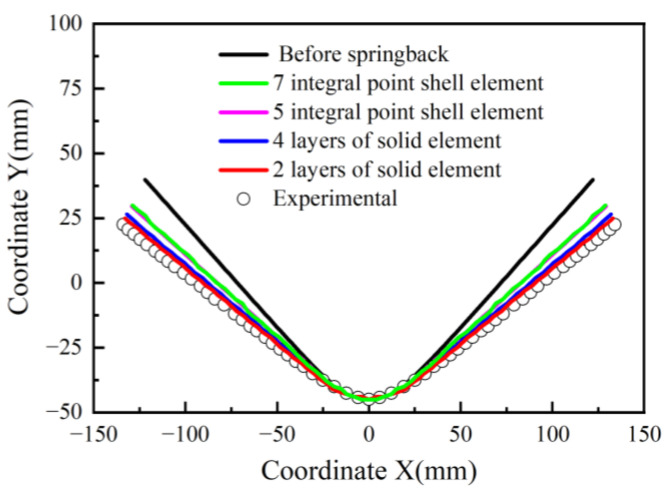
The experimental and simulated three-point bending spring-back results.

As shown in Figure 11, the spring-back result of the solid elements model (two or four layers) with the Yld2000-3d yield function agrees better with the experimental results than that of the shell elements (five or seven integral points) with the Yld2000-2d yield function. This is because when using the solid elements in the simulations, the Yld2000-3d three-dimensional stress yield function comprehensively considers the normal stress and shear stress of the sheet thickness direction. The accuracy of the new model for capturing the anisotropy of the 5754O aluminum alloy in the 3D stress space is further verified. There is no obvious difference between the two simulated results based on four layers and two layers of solid element, or the two simulated results based on five and seven integral points shell elements.

### 3.4. DP980 Steel Plate Anisotropy Prediction Verification

In this section, the ability of the extended 3D model (Yld2000-3d) to capture the anisotropy of the rest of the plates and processing is further tested by numerical simulation of cylindrical deep drawing tests on DP980 plates, in the hope that the study of 5754O aluminum alloy plates will provide a new idea for the description of the anisotropic behavior of metal sheet materials. The material properties and the cylindrical deep drawing test of DP980 steel sheets given by Cai et al. [40] were adopted. The dimensions of the tools for the deep drawing tests are specified as follows: the punch diameter is 50.00 mm, the punch profile radius is 5.00 mm, the opening diameter of the die is 53.64 mm, and the die profile radius is 13.00 mm. The thickness of the DP980 steel sheet used in the test is 1.2 mm. The blank holding forces (BHF) used in the deep drawing tests are 15 kN equally, and the Coulomb coefficient of friction is set to be 0.2 in the simulations. The stamping processing speed is 20 mm/min. Mechanical properties of DP980 steel alloy sheet are shown in Table 3.

**Figure 12:**
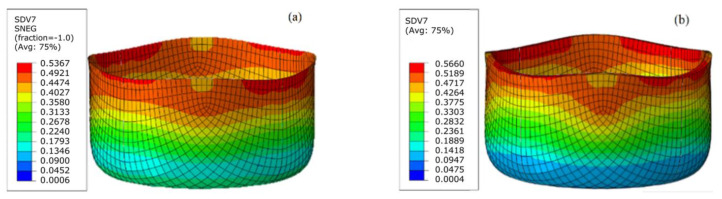
Cylindrical deep drawing strain of DP980 steel alloy sheets with shell and solid elements: (**a**) S4R shell elements (Yld2000-2d); (**b**) C3D8R solid elements (Yld20000-3d).

As before, in the FEM simulations, the two-dimensional S4R shell elements and three-dimensional C3D8R solid elements are used. The dies are established with discrete rigid elements. The simulated profiles of the deep drawing test are shown in Figure 12.

**Figure 13:**
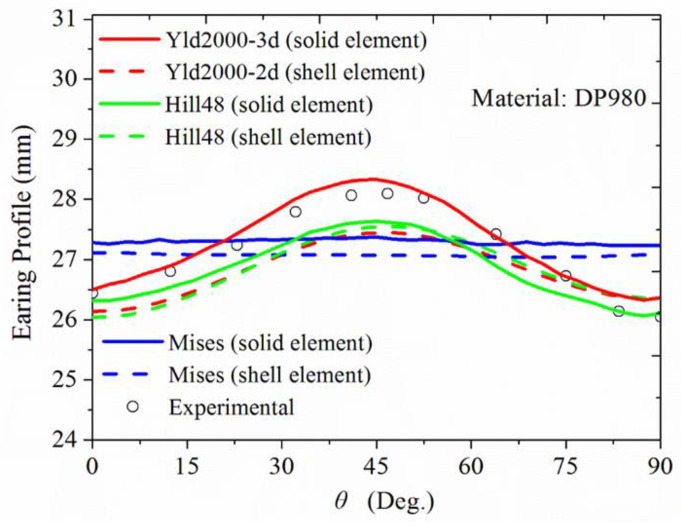
Experimental and simulation results of DP980 cylindrical deep drawing earring profile with different yield functions.

The comparison between the experimental and numerical predicted earring profiles is presented in Figure 13.

As shown in Figure 13, the simulated results based on theYld2000-3d yield function and three-dimensional C3D8R solid elements are in better agreement with the experimental results than other yield functions. Although the Hill’s quadratic and Yld2000-2d yield function could predict the locations of the peaks and valleys, the amplitudes of the earring profiles are underestimated. Since no anisotropy is considered, the Mises yield function could not predict the earring phenomenon. The Yld2000-3d yield function could provide precise predictions of the peaks’ heights, and relatively reasonable estimates of the valleys’ heights, indicating that the model generalizes for the remaining plates in characterizing the anisotropy.

## 4. Conclusions

The uniaxial tensile test, biaxial tensile test, and three-point bending models of the aluminum alloy were sequentially conducted and compared with finite element simulations to verify the accuracy of the model extended to a three-dimensional stress state (Yld2000-3d), which captures the anisotropic mechanical properties of the 5754O aluminum alloy with greater precision. The model was also used to test the rest of the plates and different loading methods, further extending the generalization of the model to different materials and deformation processes. We will continue to test the model for more complex deformation modes or new materials in the following work.

(1) Considering that some actual sheet metal forming processes are under a three-dimensional stress condition, the Yld2000-2d anisotropic yield function is extended into three-dimensional space for hydrostatic-stress-independent materials; this extended function is named the Yld2000-3d yield function. Taking the 5754O aluminum sheet as an example, the yield surface and the plastic strain ratio diagram are drawn based on the Yld2000-3d three-dimensional stress yield function developed in this study. The results show that the developed Yld2000-3d anisotropic yield function can accurately describe the uniaxial and biaxial mechanical properties of 5754O aluminum alloy sheets, and it can predict the mechanical behaviors in YZ and XZ planes.
(2) The Yld2000-3d function developed in this study is implemented in ABAQUS software by using the UMAT (VUMAT) user subroutine. The three-points bending test of a 5754O aluminum alloy sheet is carried out and the corresponding FEM simulations are performed based on the Yld2000-2d and Yld2000-3d yield functions. It is shown that the simulated results of three-points bending based on the Yld2000-3d yield function with a solid element agree better with the experimental results than those based on the Yld2000-2d yield function with a shell element.
(3) Further testing of other sheet metal forming processes was carried out by simulating deep drawing tests of DP980 steel plates using the Yld2000-3d, Yld2000-2d, Hill48, and Mises yield functions. The comparison between the simulated and experimental results of the deep drawing test shows that the Yld2000-3d yield function has the highest accuracy among them in predicting the earring profile.
(4) Besides the advantages of the Yld2000-2d yield function, the stress components related to the thickness direction are also considered, and the Yld2000-3d yield function has high accuracy in describing the anisotropic behavior under a 3D stress state. Compared to the Yld2000-2d yield function, there is no additional parameter in the Yld2000-3d yield function, which makes it convenient in engineering applications.

## Figures and Tables

**Table 1:** Mechanical properties of 5754O aluminum alloy sheets.

Loading Direction to the Rolling Direction	Yield Stress (MPa)	r Value
0°	108.7	0.707
45°	108.7	0.894
90°	113.4	0.956

**Table 2:** Biaxial mechanical properties of 5754O aluminum alloy sheets.

Loading Ratios	Yield Stress Ratios	$\beta =\arctan(d\varepsilon_{y}/d\varepsilon_{x})$(o)
$\sigma_{x}:\sigma_{y}=4:0$	$\sigma_{x}:\bar{\sigma }=1$ $\sigma_{y}:\bar{\sigma }=0$	−22.5
$\sigma_{x}:\sigma_{y}=4:1$	$\sigma_{x}:\bar{\sigma }=1.08$ $\sigma_{y}:\bar{\sigma }=0.28$	−8.25
$\sigma_{x}:\sigma_{y}=4:2$	$\sigma_{x}:\bar{\sigma }=1.09$ $\sigma_{y}:\bar{\sigma }=0.55$	−0.13
$\sigma_{x}:\sigma_{y}=4:3$	$\sigma_{x}:\bar{\sigma }=1.10$ $\sigma_{y}:\bar{\sigma }=0.88$	7.93
$\sigma_{x}:\sigma_{y}=4:4$	$\sigma_{x}:\bar{\sigma }=1.01$ $\sigma_{y}:\bar{\sigma }=1.01$	47.21
$\sigma_{x}:\sigma_{y}=1:4$	$\sigma_{x}:\bar{\sigma }=0.85$ $\sigma_{y}:\bar{\sigma }=1.12$	98.18
$\sigma_{x}:\sigma_{y}=2:4$	$\sigma_{x}:\bar{\sigma }=0.59$ $\sigma_{y}:\bar{\sigma }=1.14$	89.67
$\sigma_{x}:\sigma_{y}=3:4$	$\sigma_{x}:\bar{\sigma }=0.3$ $\sigma_{y}:\bar{\sigma }=1.13$	82.69
$\sigma_{x}:\sigma_{y}=0:4$	$\sigma_{x}:\bar{\sigma }=0$ $\sigma_{y}:\bar{\sigma }=1.04$	116.05

**Table 3:** Mechanical properties of DP980 steel alloy sheets.

Materials	Direction from RD (deg)	$\sigma_{0.2}$(MPa)	$\sigma_{b}$(MPa)	r-Value
DP980	0	706.7	1023	0.609
	45	685.6	991	0.914
	90	724.9	1048	0.749

## Data Availability

Data are contained within the article.

## Appendix A

At the analysis step “*n*” in the FEM simulation, the strain increment within the local material coordinate is as follows:

(A1) $$\Delta \varepsilon ={\left[\begin{array}{cccccc} \Delta \varepsilon_{x} & \Delta \varepsilon_{y} & \Delta \varepsilon_{z} & \Delta \varepsilon_{\mathrm{xy}} & \Delta \varepsilon_{\mathrm{xz}} & \Delta \varepsilon_{yz} \end{array}\right]}^{T}$$

Then,

(A2) $$\bar{\sigma }(\sigma_{n+1})=\bar{\sigma }(\sigma_{n}+\Delta \sigma_{n+1})=\rho$$

(A3) $$\Delta \sigma_{n+1}=\sigma_{n+1}-\sigma_{n}=C(\Delta \varepsilon -\Delta \varepsilon_{n+1}^{p})$$

(A4) $$\rho =\rho ({\bar{\varepsilon }}_{n+1}^{p})=\rho ({\bar{\varepsilon }}_{n}^{p}+\Delta {\bar{\varepsilon }}_{n+1}^{p})$$

(A5) $$\Delta \varepsilon_{n+1}^{p}=\Delta {\bar{\varepsilon }}_{n+1}^{p}\frac{\partial \bar{\sigma }}{\partial \sigma_{n+1}}$$

In Equation (A4), $\bar{\sigma }$ and ${\bar{\varepsilon }}^{p}$ represent the equivalent stress and equivalent plastic strain, respectively; $\varepsilon^{p}$ is the tensor of the plastic strain; C is the fourth-order tensor of elastic modulus; ρ is the hardening curve, and the subscript represents the analysis step; and σ and ε are the three-dimensional stress and strain, respectively, where

(A6) $$\sigma ={\left[\begin{array}{cccccc} \sigma_{x} & \sigma_{y} & \sigma_{z} & \sigma_{xy} & \sigma_{xz} & \sigma_{yz} \end{array}\right]}^{T}$$

(A7) $$\varepsilon ={\left[\begin{array}{cccccc} \varepsilon_{x} & \varepsilon_{y} & \varepsilon_{z} & \varepsilon_{\mathrm{xy}} & \varepsilon_{\mathrm{xz}} & \varepsilon_{yz} \end{array}\right]}^{T}$$

During the stress updating process, with regard to a certain three-dimensional strain increment Δε, assume the updated stress as the elastic stress firstly, and we have:

(A8) $$\sigma_{n+1}^{t}=\sigma_{n}+C\Delta \varepsilon$$

where the superscript *t* represents an attempting condition, and the plastic variable is the same as the previous value.

(A9) $${\bar{\varepsilon }}_{n+1}^{pt}={\bar{\varepsilon }}_{n}^{p}$$

If the given elastic tolerance is satisfied with regard to the following condition, then the step “*n* + 1” is the elastic condition:

(A10) $$\bar{\sigma }(\sigma_{n+1}^{t})-\rho ({\bar{\varepsilon }}_{n+1}^{pt})<Tol^{e}$$

If the above-mentioned condition is not satisfied, then this step is the elastic-plastic stage. Set the above attempting condition as the starting value of the plastic adjusting program. The new attempting stress should be on the new yield surface, that is:

(A11) $$\sigma_{n+1}=\sigma_{n+1}^{t}-C\Delta \varepsilon_{n+1}^{p}=\sigma_{n+1}^{t}-C\Delta {\bar{\varepsilon }}_{n+1}^{p}\frac{\partial \bar{\sigma }}{\partial \sigma_{n+1}}$$

The consistency requirements need to be satisfied on the new yield surface, that is:

(A12) $$\bar{\sigma }(\sigma_{n+1})-\rho ({\bar{\varepsilon }}_{n+1}^{p})=\bar{\sigma }(\sigma_{n+1}^{t}-C\Delta {\bar{\varepsilon }}_{n+1}^{p}\frac{\partial \bar{\sigma }}{\partial \sigma_{n+1}})-\rho ({\bar{\varepsilon }}_{n}^{p}+\Delta {\bar{\varepsilon }}_{n+1}^{p})=0$$

Equation (A12) is the nonlinear equation to solve the equivalent plastic strain increment $\Delta {\bar{\varepsilon }}_{n+1}^{p}$. The prediction-adjusting system based on Newton–Raphson method is adopted to solve $\Delta {\bar{\varepsilon }}_{n+1}^{p}$. Each three-dimensional tensor in this system is updated with each update of $\Delta {\bar{\varepsilon }}_{n+1}^{p}$.

When calculating $\Delta {\bar{\varepsilon }}_{n+1}^{p}$ with the Newton–Raphson method, $\frac{\partial \phi }{\partial \sigma }$ and $\frac{\partial^{2}\phi }{\partial \sigma \partial \sigma }$ need to be calculated. The expressions of $\frac{\partial \phi }{\partial \sigma }$ and $\frac{\partial^{2}\phi }{\partial \sigma \partial \sigma }$ will be given in Appendix B and Appendix C.

In this study, the solving method of the tangent modulus that is used in the literature is adopted, and the tangent modulus ${\bar{C}}^{ep}$ that is suitable for the three-dimensional stress yield function is deduced:

(A13) $${\bar{C}}^{ep}=\bar{C}-\frac{\bar{C}\frac{\partial \bar{\sigma }}{\partial \sigma_{n+1}}⊗\bar{C}\frac{\partial \bar{\sigma }}{\partial \sigma_{n+1}}}{\frac{\partial \bar{\sigma }}{\partial \sigma_{n+1}}\bar{C}\frac{\partial \bar{\sigma }}{\partial \sigma_{n+1}}+H}$$

## Appendix B

The three-dimensional “Yld2000-3d” yield function is expressed as follows:

(A14) $$\phi =\phi^{\prime }+\phi^{\prime \prime }=2{\bar{\sigma }}^{m}$$

(A15) $$\phi^{\prime }={\left|X_{1}^{\prime }-X_{2}^{\prime }\right|}^{m},\phi^{\prime \prime }={\left|2X_{2}^{\prime \prime }+X_{1}^{\prime \prime }\right|}^{m}+{\left|2X_{1}^{\prime \prime }+X_{2}^{\prime \prime }\right|}^{m}$$

(A16) $$\{\begin{array}{c} X_{1}^{\prime }=\frac{1}{2}(X_{11}^{\prime }+X_{22}^{\prime }+\sqrt{{\left(X_{11}^{\prime }-X_{22}^{\prime }\right)}^{2}+4{{\tilde{X}}^{\prime }}^{2}})\\ X_{2}^{\prime }=\frac{1}{2}(X_{11}^{\prime }+X_{22}^{\prime }-\sqrt{{\left(X_{11}^{\prime }-X_{22}^{\prime }\right)}^{2}+4{{\tilde{X}}^{\prime }}^{2}})\\ X_{1}^{\prime \prime }=\frac{1}{2}(X_{11}^{\prime \prime }+X_{22}^{\prime \prime }+\sqrt{{\left(X_{11}^{\prime \prime }-X_{22}^{\prime \prime }\right)}^{2}+4{{\tilde{X}}^{\prime \prime }}^{2}})\\ X_{2}^{\prime \prime }=\frac{1}{2}(X_{11}^{\prime \prime }+X_{22}^{\prime \prime }-\sqrt{{\left(X_{11}^{\prime \prime }-X_{22}^{\prime \prime }\right)}^{2}+4{{\tilde{X}}^{\prime \prime }}^{2}}) \end{array}$$

where

(A17) $${{\tilde{X}}^{\prime }}^{2}={X_{12}^{\prime }}^{2}+{X_{23}^{\prime }}^{2}+{X_{13}^{\prime }}^{2}$$

(A18) $${{\tilde{X}}^{\prime \prime }}^{2}={X_{12}^{\prime \prime }}^{2}+{X_{23}^{\prime \prime }}^{2}+{X_{13}^{\prime \prime }}^{2}$$

The elements in $X^{\prime }$ and $X^{\prime \prime }$ are converted by Cauchy stress:

(A19) $$X^{\prime }=L^{\prime }\sigma ,\;X^{\prime \prime }=L^{\prime \prime }\sigma$$

(A20) $$\left[\begin{array}{c} L_{11}^{\prime }\\ L_{12}^{\prime }\\ L_{21}^{\prime }\\ L_{22}^{\prime }\\ L_{66}^{\prime } \end{array}\right]=\left[\begin{array}{ccc} 2/3 & 0 & 0\\ -1/3 & 0 & 0\\ 0 & -1/3 & 0\\ 0 & 2/3 & 0\\ 0 & 0 & 1 \end{array}\right]\cdot \left[\begin{array}{c} \beta_{1}\\ \beta_{2}\\ \beta_{7} \end{array}\right],\;\left[\begin{array}{c} L_{11}^{\prime \prime }\\ L_{12}^{\prime \prime }\\ L_{21}^{\prime \prime }\\ L_{22}^{\prime \prime }\\ L_{66}^{\prime \prime } \end{array}\right]=\frac{1}{9}\cdot \left[\begin{array}{ccccc} -2 & 2 & 8 & -2 & 0\\ 1 & -4 & -4 & 4 & 0\\ 4 & -4 & -4 & 1 & 0\\ -2 & 8 & 2 & -2 & 0\\ 0 & 0 & 0 & 0 & 9 \end{array}\right]\cdot \left[\begin{array}{c} \beta_{3}\\ \beta_{4}\\ \beta_{5}\\ \beta_{6}\\ \beta_{8} \end{array}\right]$$

The parameters are determined by the following method:

(A21) $$F=\phi -2{\left(\bar{\sigma }/\sigma \right)}^{m}=0$$

(A22) $$G=q_{x}\frac{\partial \phi }{\partial s_{xx}}-q_{y}\frac{\partial \phi }{\partial s_{yy}}=0$$

(A23) $$\begin{array}{l} \phi ={\left|\beta_{1}\gamma -\beta_{2}\delta \right|}^{m}+{\left|\beta_{3}\gamma +2\beta_{4}\delta \right|}^{m}+\\ {\left|2\beta_{5}\gamma +\beta_{6}\delta \right|}^{m}-2{\left(\bar{\sigma }/\sigma \right)}^{m}=0 \end{array}$$

(A24) $$\begin{array}{l} F={\left|\frac{\sqrt{k_{2}^{\prime 2}+4\beta_{7}^{2}}}{2}\right|}^{m}+{\left|\frac{3k_{1}^{\prime \prime }-\sqrt{k_{2}^{\prime \prime 2}+4\beta_{8}^{2}}}{4}\right|}^{m}+\\ {\left|\frac{3k_{1}^{\prime \prime }+\sqrt{k_{2}^{\prime \prime 2}+4\beta_{8}^{2}}}{2}\right|}^{m}=2{\left(\bar{\sigma }/\sigma_{45}\right)}^{m} \end{array}$$

(A25) $$G=\frac{\partial \phi }{\partial \sigma_{xx}}+\frac{\partial \phi }{\partial \sigma_{yy}}-\frac{2m{\bar{\sigma }}^{m}}{\sigma_{45}(1+r_{45})}=0$$

In each of the above equations:

(A26) s=Tσ

(A27) $$T=\left[\begin{array}{ccc} 2/3 & -1/3 & 0\\ -1/3 & 2/3 & 0\\ 0 & 0 & 1 \end{array}\right]$$

(A28) $${k^{\prime }}_{2}=\frac{\beta_{1}-\beta_{2}}{3}$$

(A29) $$k_{1}^{\prime \prime }=\frac{2\beta_{5}+\beta_{6}+\beta_{3}+2\beta_{4}}{9}$$

(A30) $$k_{2}^{\prime \prime }=\frac{2\beta_{5}+\beta_{6}-\beta_{3}-2\beta_{4}}{3}$$

The Newton–Raphson numerical procedure is used to solve for the eight $\beta_{k}$ coefficients simultaneously. The two matrices $L^{\prime }$ and $L^{\prime \prime }$ are completely defined with these eight coefficients.

The first derivative of the three-dimensional “Yld2000-3d” yield function is as follows:

(A31) $$\frac{\partial \phi }{\partial \sigma_{xx}}=\begin{array}{c} \frac{\partial \phi^{\prime }}{\partial X_{1}^{\prime }}(\frac{\partial X_{1}^{\prime }}{\partial X_{11}^{\prime }}\cdot L_{11}^{\prime }+\frac{\partial X_{1}^{\prime }}{\partial X_{22}^{\prime }}\cdot L_{21}^{\prime })+\frac{\partial \phi^{\prime }}{\partial X_{2}^{\prime }}(\frac{\partial X_{2}^{\prime }}{\partial X_{11}^{\prime }}\cdot L_{11}^{\prime }+\frac{\partial X_{2}^{\prime }}{\partial X_{22}^{\prime }}\cdot L_{21}^{\prime })\\ +\frac{\partial \phi^{\prime \prime }}{\partial X_{1}^{\prime \prime }}(\frac{\partial X_{1}^{\prime \prime }}{\partial X_{11}^{\prime \prime }}\cdot L_{11}^{\prime \prime }+\frac{\partial X_{1}^{\prime \prime }}{\partial X_{22}^{\prime \prime }}\cdot L_{21}^{\prime \prime })+\frac{\partial \phi^{\prime \prime }}{\partial X_{2}^{\prime \prime }}(\frac{\partial X_{2}^{\prime \prime }}{\partial X_{11}^{\prime \prime }}\cdot L_{11}^{\prime \prime }+\frac{\partial X_{2}^{\prime \prime }}{\partial X_{22}^{\prime \prime }}\cdot L_{21}^{\prime \prime }) \end{array}$$

(A32) $$\frac{\partial \phi }{\partial \sigma_{yy}}=\begin{array}{c} \frac{\partial \phi^{\prime }}{\partial X_{1}^{\prime }}(\frac{\partial X_{1}^{\prime }}{\partial X_{11}^{\prime }}\cdot L_{12}^{\prime }+\frac{\partial X_{1}^{\prime }}{\partial X_{22}^{\prime }}\cdot L_{22}^{\prime })+\frac{\partial \phi^{\prime }}{\partial X_{2}^{\prime }}(\frac{\partial X_{2}^{\prime }}{\partial X_{11}^{\prime }}\cdot L_{12}^{\prime }+\frac{\partial X_{2}^{\prime }}{\partial X_{22}^{\prime }}\cdot L_{22}^{\prime })\\ +\frac{\partial \phi^{\prime \prime }}{\partial X_{1}^{\prime \prime }}(\frac{\partial X_{1}^{\prime \prime }}{\partial X_{11}^{\prime \prime }}\cdot L_{12}^{\prime \prime }+\frac{\partial X_{1}^{\prime \prime }}{\partial X_{22}^{\prime \prime }}\cdot L_{22}^{\prime \prime })+\frac{\partial \phi^{\prime \prime }}{\partial X_{2}^{\prime \prime }}(\frac{\partial X_{2}^{\prime \prime }}{\partial X_{11}^{\prime \prime }}\cdot L_{12}^{\prime \prime }+\frac{\partial X_{2}^{\prime \prime }}{\partial X_{22}^{\prime \prime }}\cdot L_{22}^{\prime \prime }) \end{array}$$

(A33) $$\frac{\partial \phi }{\partial \sigma_{zz}}=\begin{array}{c} \begin{array}{l} \frac{\partial \phi^{\prime }}{\partial X_{1}^{\prime }}(\frac{\partial X_{1}^{\prime }}{\partial X_{11}^{\prime }}\cdot (-L_{11}^{\prime }+L_{12}^{\prime })+\frac{\partial X_{1}^{\prime }}{\partial X_{22}^{\prime }}\cdot (-L_{21}^{\prime }+L_{22}^{\prime }))+\frac{\partial \phi^{\prime }}{\partial X_{2}^{\prime }}(\frac{\partial X_{2}^{\prime }}{\partial X_{11}^{\prime }}\cdot (-L_{11}^{\prime }+L_{12}^{\prime })+\\ \frac{\partial X_{2}^{\prime }}{\partial X_{22}^{\prime }}\cdot (-L_{21}^{\prime }+L_{22}^{\prime }))+\frac{\partial \phi^{\prime \prime }}{\partial X_{1}^{\prime \prime }}(\frac{\partial X_{1}^{\prime \prime }}{\partial X_{11}^{\prime \prime }}\cdot (-L_{11}^{\prime \prime }+L_{12}^{\prime \prime })+\frac{\partial X_{1}^{\prime \prime }}{\partial X_{22}^{\prime \prime }}\cdot (-L_{21}^{\prime \prime }+L_{22}^{\prime \prime }))+ \end{array}\\ \frac{\partial \phi^{\prime \prime }}{\partial X_{2}^{\prime \prime }}(\frac{\partial X_{2}^{\prime \prime }}{\partial X_{11}^{\prime \prime }}\cdot (-L_{11}^{\prime \prime }+L_{12}^{\prime \prime })+\frac{\partial X_{2}^{\prime \prime }}{\partial X_{22}^{\prime \prime }}\cdot (-L_{21}^{\prime \prime }+L_{22}^{\prime \prime })) \end{array}$$

(A34) $$\frac{\partial \phi }{\partial \sigma_{xy}}=(\frac{\partial \phi^{\prime }}{\partial X_{1}^{\prime }}\frac{\partial X_{1}^{\prime }}{\partial X_{12}^{\prime }}+\frac{\partial \phi^{\prime }}{\partial X_{2}^{\prime }}\frac{\partial X_{2}^{\prime }}{\partial X_{12}^{\prime }})\cdot L_{66}^{\prime }+\frac{\partial \phi^{\prime \prime }}{\partial X_{1}^{\prime \prime }}\frac{\partial X_{1}^{\prime \prime }}{\partial X_{12}^{\prime \prime }}+\frac{\partial \phi^{\prime \prime }}{\partial X_{2}^{\prime \prime }}\frac{\partial X_{2}^{\prime \prime }}{\partial X_{12}^{\prime \prime }})\cdot L_{66}^{\prime \prime }$$

(A35) $$\frac{\partial \phi }{\partial \sigma_{xz}}=(\frac{\partial \phi^{\prime }}{\partial X_{1}^{\prime }}\frac{\partial X_{1}^{\prime }}{\partial X_{13}^{\prime }}+\frac{\partial \phi^{\prime }}{\partial X_{2}^{\prime }}\frac{\partial X_{2}^{\prime }}{\partial X_{13}^{\prime }})\cdot L_{66}^{\prime }+\frac{\partial \phi^{\prime \prime }}{\partial X_{1}^{\prime \prime }}\frac{\partial X_{1}^{\prime \prime }}{\partial X_{13}^{\prime \prime }}+\frac{\partial \phi^{\prime \prime }}{\partial X_{2}^{\prime \prime }}\frac{\partial X_{2}^{\prime \prime }}{\partial X_{13}^{\prime \prime }})\cdot L_{66}^{\prime \prime }$$

(A36) $$\frac{\partial \phi }{\partial \sigma_{yz}}=(\frac{\partial \phi^{\prime }}{\partial X_{1}^{\prime }}\frac{\partial X_{1}^{\prime }}{\partial X_{23}^{\prime }}+\frac{\partial \phi^{\prime }}{\partial X_{2}^{\prime }}\frac{\partial X_{2}^{\prime }}{\partial X_{23}^{\prime }})\cdot L_{66}^{\prime }+\frac{\partial \phi^{\prime \prime }}{\partial X_{1}^{\prime \prime }}\frac{\partial X_{1}^{\prime \prime }}{\partial X_{23}^{\prime \prime }}+\frac{\partial \phi^{\prime \prime }}{\partial X_{2}^{\prime \prime }}\frac{\partial X_{2}^{\prime \prime }}{\partial X_{23}^{\prime \prime }})\cdot L_{66}^{\prime \prime }$$

## Appendix C

Because the $\frac{\partial \phi }{\partial \sigma }$ expression in B complicated, the $\frac{\partial^{2}\phi }{\partial \sigma \partial \sigma }$ expression is lengthier. Therefore, the second derivate expression of each stress component is not given in detail. The overall expression of $\frac{\partial^{2}\phi }{\partial \sigma \partial \sigma }$ is given here. In order to express it conveniently, the improved yield function is simply converted as follows:

(A37) $$\phi =\phi^{\prime }+\phi^{\prime \prime }=2{\bar{\sigma }}^{m}$$

(A38) $$\phi^{\prime }={\left|Y_{1}^{\prime }-Y_{2}^{\prime }\right|}^{m},\phi^{\prime \prime }={\left|2Y_{2}^{\prime \prime }+Y_{1}^{\prime \prime }\right|}^{m}+{\left|2Y_{1}^{\prime \prime }+Y_{2}^{\prime \prime }\right|}^{m}$$

where

(A39) $$\{\begin{array}{c} Y_{1}^{\prime }=\frac{1}{2}(X_{1}^{\prime }+X_{2}^{\prime }+\sqrt{{\left(X_{1}^{\prime }-X_{2}^{\prime }\right)}^{2}+4{X_{3}^{\prime }}^{2}})\\ Y_{2}^{\prime }=\frac{1}{2}(X_{1}^{\prime }+X_{2}^{\prime }-\sqrt{{\left(X_{1}^{\prime }-X_{2}^{\prime }\right)}^{2}+4{X_{3}^{\prime }}^{2}})\\ Y_{1}^{\prime \prime }=\frac{1}{2}(X_{1}^{\prime \prime }+X_{2}^{\prime \prime }+\sqrt{{\left(X_{1}^{\prime \prime }-X_{2}^{\prime \prime }\right)}^{2}+4{X_{3}^{\prime \prime }}^{2}})\\ Y_{2}^{\prime \prime }=\frac{1}{2}(X_{1}^{\prime \prime }+X_{2}^{\prime \prime }-\sqrt{{\left(X_{1}^{\prime \prime }-X_{2}^{\prime \prime }\right)}^{2}+4{X_{3}^{\prime \prime }}^{2}}) \end{array}$$

(A40) $${X_{3}^{\prime }}^{2}={X_{12}^{\prime }}^{2}+{X_{23}^{\prime }}^{2}+{X_{13}^{\prime }}^{2},\;{X_{3}^{\prime \prime }}^{2}={X_{12}^{\prime \prime }}^{2}+{X_{23}^{\prime \prime }}^{2}+{X_{13}^{\prime \prime }}^{2}$$

The second derivate of the “Yld2000-3d” three-dimensional stress yield function is as follows:

(A41) $$\frac{\partial^{2}\phi }{\partial \sigma_{i}\sigma_{j}}=\begin{array}{c} \sum_{a}^{2}\sum_{b}^{3}\sum_{c}^{2}\sum_{d}^{3}\left\{\frac{\partial^{2}\phi^{\prime }}{\partial Y_{a}^{\prime }\partial Y_{c}^{\prime }}(\frac{\partial Y_{a}^{\prime }}{\partial X_{b}^{\prime }}\frac{\partial X_{b}^{\prime }}{\partial \sigma_{i}})(\frac{\partial Y_{c}^{\prime }}{\partial X_{d}^{\prime }}\frac{\partial X_{d}^{\prime }}{\partial \sigma_{j}})+\frac{\partial^{2}\phi^{\prime \prime }}{\partial Y_{a}^{\prime \prime }\partial Y_{c}^{\prime \prime }}(\frac{\partial Y_{a}^{\prime \prime }}{\partial X_{b}^{\prime \prime }}\frac{\partial X_{b}^{\prime \prime }}{\partial \sigma_{i}})(\frac{\partial Y_{c}^{\prime \prime }}{\partial X_{d}^{\prime \prime }}\frac{\partial X_{d}^{\prime \prime }}{\partial \sigma_{j}})\right\}\\ +\sum_{c}^{2}\sum_{a}^{2}\sum_{d}^{2}\left\{\frac{\partial \phi^{\prime }}{\partial Y_{c}^{\prime }}\frac{\partial^{2}Y_{c}^{\prime }}{\partial X_{a}^{\prime }\partial X_{d}^{\prime }}(\frac{\partial X_{a}^{\prime }}{\partial \sigma_{i}})(\frac{\partial X_{d}^{\prime }}{\partial \sigma_{j}})+\frac{\partial \phi^{\prime \prime }}{\partial Y_{c}^{\prime \prime }}\frac{\partial^{2}Y_{c}^{\prime \prime }}{\partial X_{a}^{\prime \prime }\partial X_{d}^{\prime \prime }}(\frac{\partial X_{a}^{\prime \prime }}{\partial \sigma_{i}})(\frac{\partial X_{d}^{\prime \prime }}{\partial \sigma_{j}})\right\}\\ +\sum_{c}^{2}\sum_{d}^{3}\left\{\frac{\partial \phi^{\prime }}{\partial Y_{c}^{\prime }}\frac{\partial Y_{c}^{\prime }}{\partial X_{d}^{\prime }}(\frac{\partial^{2}X_{d}^{\prime }}{\partial \sigma_{i}\partial \sigma_{j}})+\frac{\partial \phi^{\prime \prime }}{\partial Y_{c}^{\prime \prime }}\frac{\partial^{2}Y_{c}^{\prime \prime }}{\partial X_{d}^{\prime \prime }}(\frac{\partial^{2}X_{d}^{\prime \prime }}{\partial \sigma_{i}\partial \sigma_{j}})\right\} \end{array}$$
